# Iron-related protein in prediction of acute kidney injury in the pediatric population after cardiac surgery

**DOI:** 10.1186/s13019-025-03688-0

**Published:** 2025-11-28

**Authors:** Yu Zhu, Yan-Ze Liu, Hong-Chao Wang, Zhi Geng, Yu Kan, Xu Cao, Xiang-Ming Yan

**Affiliations:** 1 Children’s Hospital of Soochow University, Soochow, China; 2 The First affiliated Hospital of Xinjiang Medical University, Urumqi, China

**Keywords:** Ferritin, Transferrin, Hepcidin, Acute kidney injury, Biomarker, Pediatric population

## Abstract

**Background:**

Acute Kidney Injury (AKI) is a frequent post-operative complication following cardiac surgery in the clinical setting. Iron metabolism disorders are a key cause of kidney injury during the ischemia-reperfusion (IR) process. However, the role of iron metabolites in prediction of AKI remains unidentified. This study investigates the roles of iron metabolites ferritin, transferrin, and hepcidin in prediction of the occurrence and severity of AKI in children following cardiac surgery.

**Methods:**

Serum and urine samples were collected from 219 children with the age from 1 to 36 months undergoing cardiopulmonary bypass (CPB), both preoperatively and postoperatively at 0 h and 6 h. Serum ferritin, urinary transferrin, and urinary hepcidin levels were measured.

**Results:**

Among the included 219 children, 66 developed AKI postoperatively. Serum ferritin, urinary transferrin, and urinary hepcidin were significantly associated with the occurrence of AKI after CPB. The area under the receiver operating characteristic curve (AUC) for serum ferritin and urinary transferrin at 6 h postoperatively, and hepcidin at 0 h postoperatively achieved optimal values (serum ferritin AUC:0.804; urinary transferrin AUC:0.805; urinary hepcidin AUC:0.894). The combined analysis of the three biomarkers yielded a higher AUC than individual biomarkers alone. The combined biomarker panel demonstrated favourable predictive performance across all AKI severity stages.

**Conclusions:**

Ferritin, transferrin, and hepcidin serve as early predictive markers for AKI associated with cardiac surgery in children and are independent predictors for postoperative AKI. These biomarkers exhibit a good early predictive capacity for the severity of AKI.

## Introduction

Congenital Heart Disease (CHD) is a common congenital disorder in children, often necessitating surgical intervention. However, cardiac surgery carries significant risks, with postoperative complications occurring in up to 43% of cases [1]. Acute Kidney Injury (AKI) is one of the most common and severe complications following pediatric cardiac surgery with cardiopulmonary bypass (CPB), with an incidence of 34.3% in some previous studies [2]. The occurrence of AKI is associated with prolonged hospital stays and ICU admissions, increased hospitalization costs, and an elevated risk of end-stage kidney disease and mortality [3]. Therefore, timely prediction and detection of postoperative AKI in cardiac surgery are imperative. Currently, serum creatinine and urine output are standard clinical markers for diagnosing AKI. However, serum creatinine has limitations in specificity and sensitivity [4,5]. The increase of serum creatinine (sCr) usually occurs on the basis of a significant reduction in glomerular filtration rate, often detectable only 48–72 h post-AKI onset, and can be influenced by non-renal factors and certain medications [4, 6]. Therefore, serum creatinine is not a perfect indicator. In recent years, numerous studies have explored early predictive markers for AKI, but they have not been widely accepted for clinical practice [7].

The role of iron metabolism in kidney diseases has long been recognized [8]. The increase of free iron and reactive oxygen species (ROS) play a crucial role in ischemia-reperfusion-induced renal injury [9–11]. It is of great significance to maintain iron homeostasis. The role of iron-related proteins in iron metabolism has been studied. Hepcidin is an iron chelator, which has demonstrated a alleviating effect in AKI by regulating iron homeostasis in an animal study [10]. Some cohort studies also proved that hepcidin has potential predictive value for postoperative AKI [12, 13]. Alfredo G et al. ‘s study showed that urine transferrin is correlated with kidney injury caused by drugs with tubular toxicity, which can identify early subclinical tubular injury in advance and has predictive significance [14, 15]. Serum ferritin though binding to free iron and limiting ROS production offers protective effects. A prospective study found that, serum ferritin and renal function recovery are positively correlated, which can be served as a prognostic marker for AKI [16]. However, the current studies about the predictive capacity of iron-related proteins for postoperative AKI in cardiac surgery are limited and more studies are aimed at adults. The kidney conditions of adults and children are different, the predictive value of iron-related proteins for AKI in children post-cardiac surgery remains unstudied. This study aims to evaluate whether serum ferritin, urinary transferrin, and urinary hepcidin could be used as early predict biomarkers for AKI following CPB in children undergoing cardiac surgery.

## Methods

### Study population

A total of 265 children aged 1 month to 3 years who underwent cardiopulmonary bypass (CPB) surgery at the Department of Cardiothoracic Surgery at the Children’s Hospital of Soochow University from August 2022 to August 2024 were included in the prospective cohort study. Exclusion criteria included: absence of clinical data or test sample collection (*n* = 37), preoperative combination of renal insufficiency or congenital renal abnormality (*n* = 2), combination of other congenital diseases (*n* = 4), and prior history of cardiac surgery (*n* = 3). This study was approved by the Ethics Committee of the Children’s Hospital of Soochow University (No.2024CS059), and informed consent was obtained from the guardians of all participants.

## Clinical and laboratory data collection

Demographic data of age, gender, height and weight of the patients were enrolled. The maximum values of serum creatinine and blood urea nitrogen (BUN) before surgery and 48 h after surgery, as well as laboratory measurement data of albumin, hemoglobin and platelet before surgery and 0 h after surgery were collected. Additionally, data on surgical duration, aortic cross-clamp time, CPB time, performance of deep hypothermic circulatory arrest (DHCA) and peritoneal dialysis, preoperative computed tomography angiography (CTA) examination, and risk adjustment for congenital heart surgery-1 (RACHS-1) score were recorded.

Blood samples and urine samples of each patient were collected at preoperative, postoperative 0 h, and 6 h intervals. These samples were centrifuged at 2500 rpm for 10 min at 4 °C, and the supernatant was stored at −80 °C.

## Definition outcomes

AKI was defined according to the Kidney Disease Improving Global Outcomes (KDIGO) criteria [17]. The baseline sCr was determined as the average of the two most recent preoperative sCr measurements. AKI was diagnosed when the sCr increased to 1.5 times or more of the baseline value or if there was an increase of more than 26.5 µmol/L within 48 h.

## Iron related factor measurement

The supernatant of blood samples was collected for the measure of ferritin, and the supernatant of urine samples was used for the measure of urinary transferrin, hepcidin and creatinine. These factors were measured by enzyme-linked immunosorbent assay (ELISA). This process was carried out in strict accordance with the manufacturer’s instructions. Urinary creatinine was performed for urinary transferrin and hepcidin concentrations to eliminate the effects of different urinary flow rates. (transferrin/uCr, hepcidin/uCr)

### Statistical analysis

Normality and homogeneity of variance tests were conducted for continuous variables. Variables following a normal distribution were expressed as mean ± standard deviation, and group differences were analyzed using the t-test. Variables not following a normal distribution were reported as median (interquartile range), and group differences were examined using the rank-sum test. Categorical variables were presented as frequency (percentage) and analyzed for differences using the Fisher’s exact test or Pearson’s test. Receiver Operating Characteristic (ROC) curves were utilized to assess the predictive ability of serum ferritin, Transferrin/uCr, and Hepcidin/uCr for AKI. The area under the ROC curve (AUC) was used to determine the efficacy of these biomarkers in predicting AKI and the severity of kidney injury. Univariate logistic regression analysis was used to explore the relationship between each biomarker and AKI, yielding unadjusted odds ratios (OR). Multivariate logistic regression analysis was performed to adjust for confounding factors, including the maximum creatinine value within 48 h postoperatively, RACHS-1 score, aortic cross-clamp time, and estimated glomerular filtration rate (eGFR) at 48 h postoperatively, providing adjusted ORs. The data were analyzed and processed by SPSS Statistical Software 25.0.and GraphPad Prism 9. and p-value < 0.05 was considered statistically significant.

## Results

### Patient characteristics

This study involved 219 children who underwent cardiothoracic CPB surgery and were admitted to the cardiothoracic surgery unit. The children were categorized into AKI group (*n* = 66) and non-AKI group (*n* = 219) according to KDIGO criteria. Based on the severity of kidney injury in AKI group, the sample were further classified into AKI stage 1 (*n* = 46), stage 2 (*n* = 18) and stage 3 (*n* = 2). In terms of patients baseline characteristics, significant differences were observed between the AKI and non-AKI groups in age (*p* < 0.001), height (*p* = 0.007), and weight (*p* = 0.001), but there were no significant differences in the gender (*p* = 0.807). We also collected baseline information about preoperative and postoperative data. Preoperatively, significant differences were noted in serum creatinine (*p* = 0.001) between the two groups, with no significant differences in other indicators. At 0 h postoperatively, several indicators showed differences compared to preoperative values. Notably, the AKI group exhibited significantly higher levels of serum creatinine (*p* = 0.001) and BUN (*p* = 0.001), and elevated hemoglobin levels (*p* = 0.045), compared to the non-AKI group, while eGFR (*p* < 0.001) was markedly decreased. During surgery, the CPB time (*p* < 0.001), aortic cross-clamp time (*p* = 0.001), and operation time (*p* < 0.001) were significantly longer for children in the AKI group compared to those in the Non-AKI group. (Table 1)

**Table 1:** Comparison of demographic and clinical characteristics between non-AKI and AKI

	Non-AKI(*n* = 153)	AKI(*n* = 66)	*P*-value
Age (mouths)	12 (4–25)	3 (1–7)	< 0.001
Sex, male, n (%)	83 (54.2)	33 (50.0)	0.807
Height (cm)	74 (62–74)	59 (52–69)	0.007
Weight (kg)	9 (6–12)	5.3 (3.8–7.9)	0.001
**Preoperative characteristics**			
CTA, n (%)	22 (14.4)	20 (30.3)	0.001
Serum creatinine umol/L	26.0 (22.2–32.2)	21.7 (19.0–24.2.0.2)	0.001
BUN mmol/L	3.8 (2.8–4.9)	3.7 (2.3–4.9)	0.674
Albumin g/L	41.7 ± 5.0	40.3 ± 6.1	0.189
Hemoglobin g/L	120 (113–128)	118.5 (104.0–128.5.0.5)	0.136
Platelet counts 10^9/L	312.0 (254.5–381.0)	321.0 (287.0–388.0.0.0)	0.581
eGFR, ml/min per 1.73 m2	117.5 (93.1–137.5.1.5)	114.5 (83.8–150.6.8.6)	0.585
**Surgical characteristics**			
CPB time (min)	70 (52–95)	81.5 (66.5–125.8.5.8)	< 0.001
Aortic cross-clamp time (min)	41 (28–63)	50 (37–72)	0.001
Surgical duration (min)	165 (140–195)	190 (151–238)	< 0.001
DHCA, n (%)	3 (2.0)	1 (1.5)	0.276
RACHS-1 score ≥ 3, n (%)	29 (19.0)	30 (45.5)	0.032
**postoperative characteristics**			
Hospital length of stay (d)	9 (8–12)	12 (10–23)	0.028
ICU length of stay (d)	2 (1–3)	5 (2–8)	0.001
Serum creatinine umol/L	24.0 (21.0–29.0)	35.0 (32.0–48.0)	0.001
BUN mmol/L	5.3 (4.2–6.6)	7.0 (5.7–9.4)	0.001
Albumin g/L	44.1 ± 2.9	41.0 ± 4.5	< 0.001
Hemoglobin g/L	104.0 (96.5–114.0)	108.0 (98.0–119.0.0.0)	0.045
Platelet counts 10^9/L	184.0 (154.5–231.0)	188.0 (151.3–235.3.3.3)	0.801
eGFR, ml/min per 1.73 m2	125.5 (101.5–149.8.5.8)	78.8 (50.4–104.5.4.5)	< 0.001
**AKI Stage**			
AKI Stage 1, n (%)	46 (19.2)		NA
AKI Stage 2, n (%)	18 (8.2)		NA
AKI Stage 3, n (%)	2 (0.9)		NA

AKI, acute kidney injury; CTA, Computed Tomography Angiography; BUN, blood urea nitrogen; eGFR, estimated glomerular filtration rate; CPB, cardiopulmonary bypass; DHCA, deep hypothermic circulatory arrest; RACHS-1, Risk Adjustment for Congenital Heart Surgery-1

## The relationship of serum ferritin, transferrin/uCr, hepcidin/uCr with AKI

### Serum ferritin

**Fig. 1:**
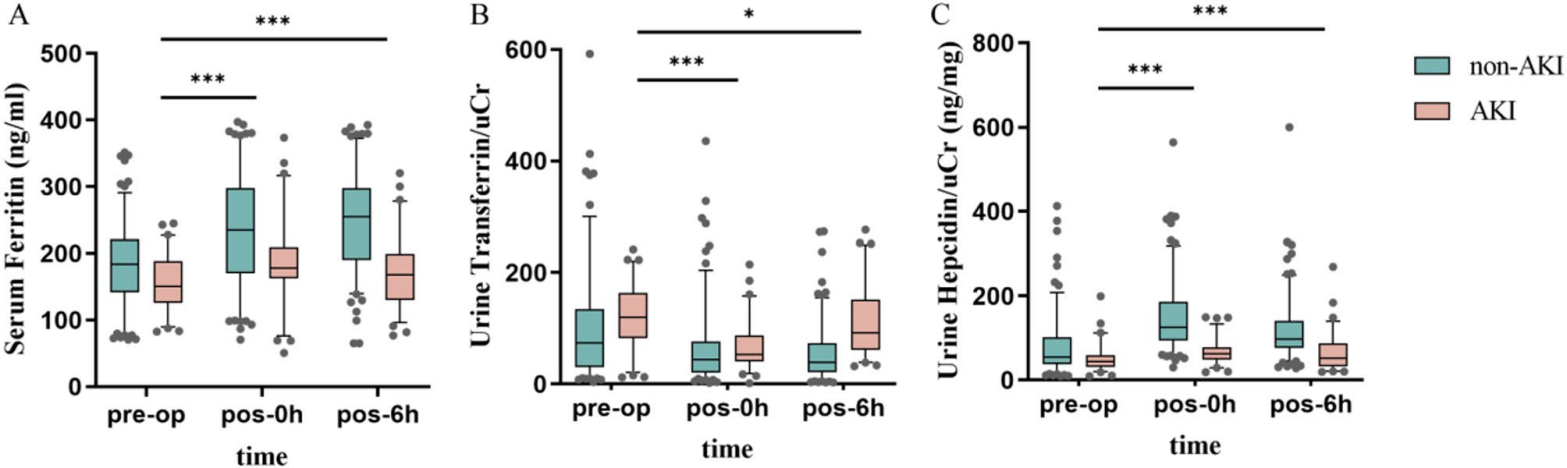
Analysis of variability among three biomarkers at different time points.Analysis of intergroup differences in serum ferritin, urine transferrin/uCr, and urine hepcidin/uCr at preoperative and postoperative 0 h and 6 h. In the AKI group, differences between preoperative and postoperative time points were statistically significant. (* *p* < 0.05,*** *p* < 0.001) **A**. analysis of intergroup differences in Serum Ferritin **B**. analysis of intergroup differences in Urine Transferrin/uCr **C**. analysis of intergroup differences in rine Hepcidin/uCr

**Fig. 2:**
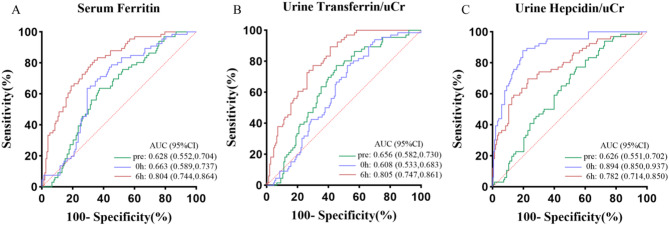
ROC analysis for a single biomarker across various time points. ROC analysis was performed to assess the predictive ability for AKI at different time points using serum ferritin, urine transferrin/uCr, and urine hepcidin/uCr. AUC, area under the curve; 95%CI, 95% Confidence intervalA.ROC analysis of serum ferritin at preoperative, 0h, and 6h postoperative time points, B.ROC analysis of urine transferrin/uCr at preoperative, 0h, and 6h postoperative time points, C.ROC analysis of urine hepcidin/uCr at preoperative, 0h, and 6h postoperative time points

**Fig. 3:**
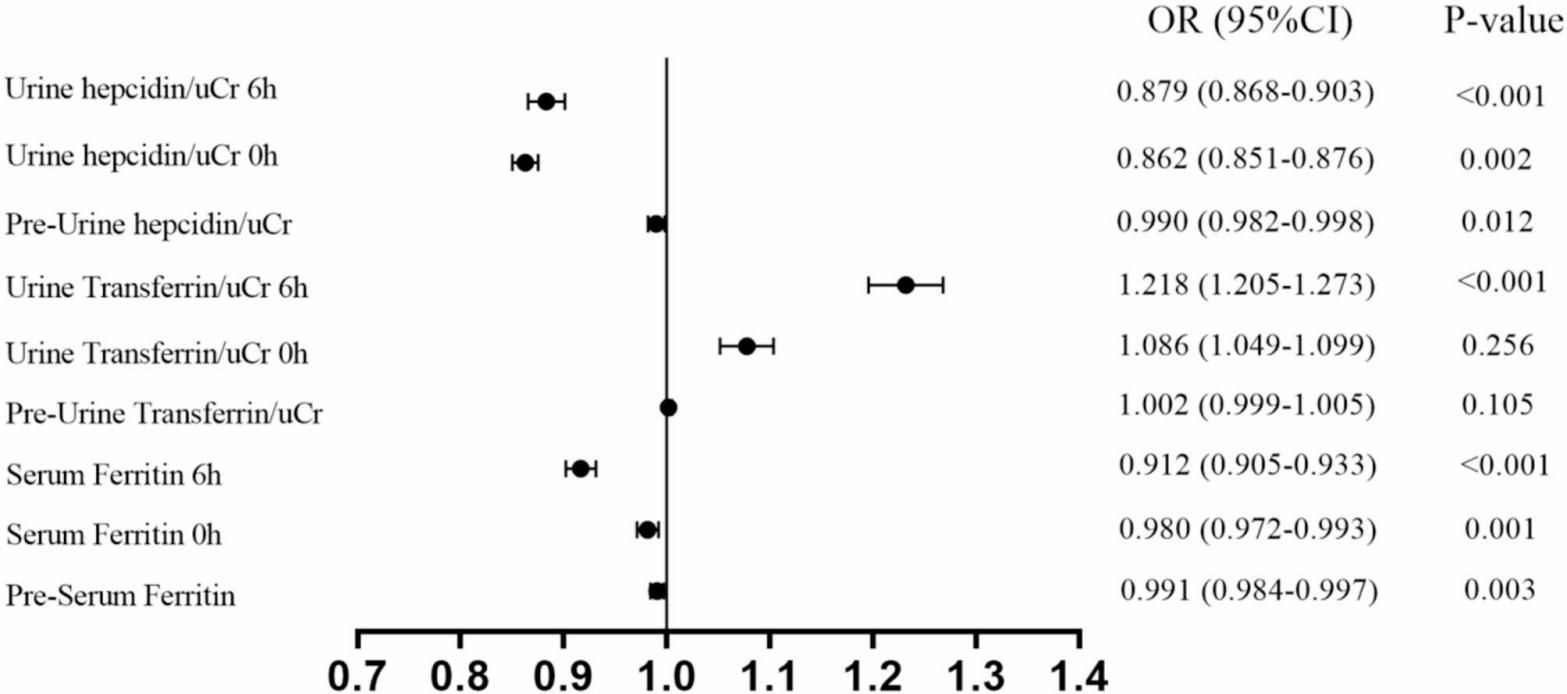
Univariate logistic regression analysis of individual biomarkers across various time points.OR: odds ratio; 95%CI, 95% Confidence interval

**Fig. 4:**
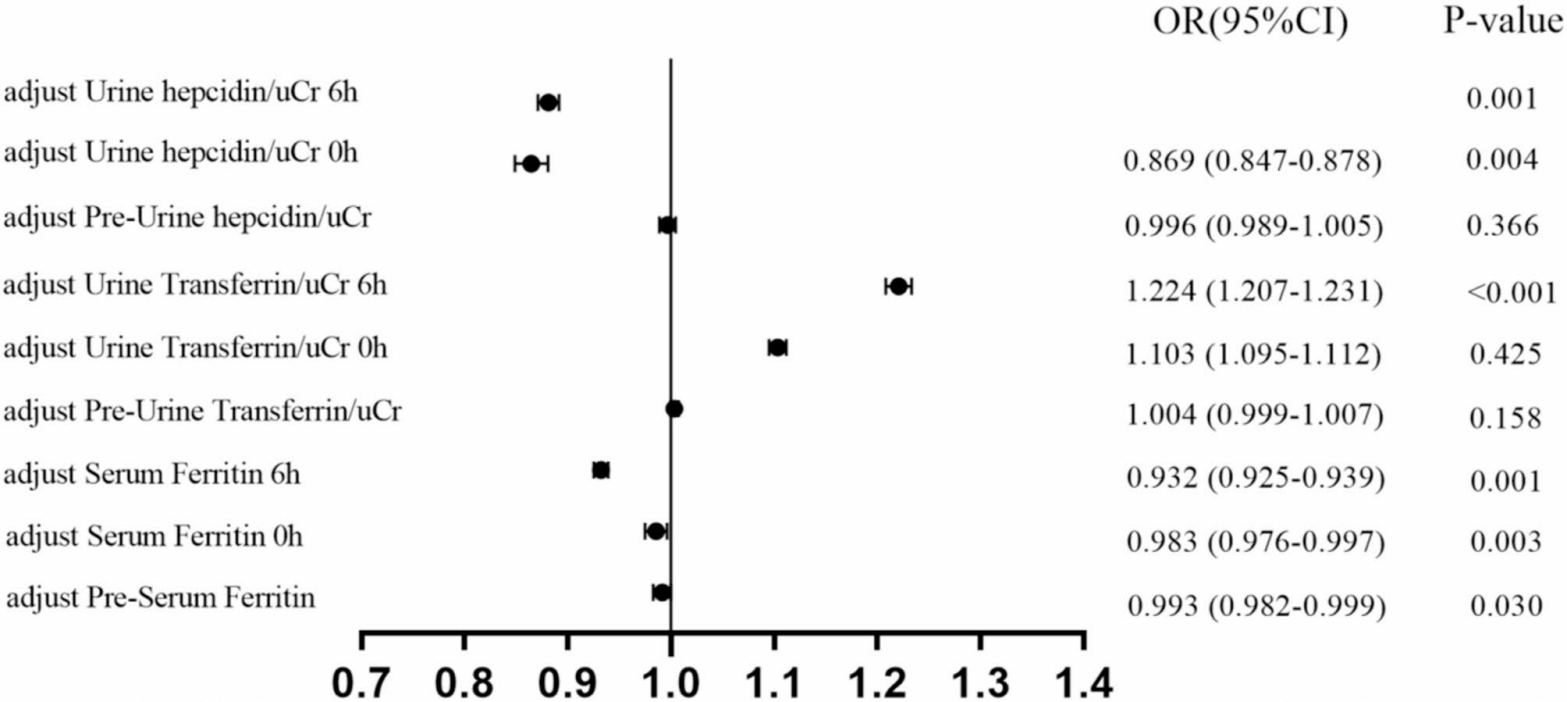
Adjusted multivariate logistic regression analysis of biomarkers considering potential confounding variables. correction indicators: age, postoperative creatinine, RACHS-1, aortic cross-clamp time, and postoperative eGFR

Preoperative and postoperative (0 h and 6 h) serum ferritin levels showed statistically significant differences between AKI and non-AKI patients, with the greatest difference observed at 6 h post-surgery (Table 2; Fig. 1A). Univariate logistic regression analysis of serum ferritin at the three time points respectively found the significant results, with the lowest odds ratio (OR) of 0.912 (0.905–0.933) and the strongest significance (*p* < 0.001) at 6 h post-surgery (Fig. 3). After adjusting for potential confounding factors with some clinical indicators, the results remained consistent (Fig. 4). The results showed that serum ferritin can serve as an independent predictor of AKI. ROC analysis of serum ferritin at different time points gained different area under the curve (AUC) values, with significant predictive value at 0 h and 6 h post-surgery. The maximum AUC (0.804) was obtained at 6 h after surgery (*p* < 0.001), showing better specificity and sensitivity and strong predictive performance (Fig. 2A). ROC analysis of perioperative changes in serum ferritin at multiple time points yielded consistent results.The largest area under the curve (AUC = 0.786, *p* < 0.001) was observed at 6 h after surgery, indicating reliable predictive performance.(Fig. 6A).

**Table 2:** Comparison of biomarker characteristics at different time points between non-AKI and AKI

	Non-AKI(*n* = 153)	AKI(*n* = 66)	*P*-value
**Preoperative biomarkers**			
Serum Ferritin (ng/ml)	182.77(141.43–221.20)	150.52(125.82–188.47.82.47)	0.048
Urine Transferrin/uCr	74.22(30.55–134.27.55.27)	120.49(82.18–163.00)	0.010
Urine Hepcidin/uCr (ng/mg)	54.80(37.84–102.35.84.35)	43.83(31.68–59.47)	0.009
**Postoperative 0 h biomarkers**			
Serum Ferritin 0 h (ng/ml)	234.20(17.09–298.20)	178.26(162.62–209.16.62.16)	< 0.001
Urine Transferrin/uCr 0 h	43.62(20.11–76.98)	53.37(40.38–87.98)	0.002
Urine Hepcidin/uCr 0 h (ng/mg)	125.66 (94.17–185.87.17.87)	63.02(48.65–78.51)	< 0.001
**Postoperative 6 h biomarkers**			
Serum Ferritin 6 h (ng/ml)	251.36(190.20–297.66.20.66)	167.70(130.40–198.82.40.82)	< 0.001
Urine Transferrin/uCr 6 h	39.50(21.12–73.10)	92.29(61.00–151.59.00.59)	0.004
Urine Hepcidin/uCr 6 h (ng/mg)	97.47(76.02–140.74.02.74)	52.54 (32.95–86.58)	0.001

### Transferrin/uCr

Analysis of transferrin/uCr levels at different time points shows a significant decrease over time in AKI and non-AKI patients, with statistically significant differences (Table 2; Fig. 1B). The univariate regression analysis of transferrin/uCr found that transferrin/uCr had the most significant OR value of 1.218 (1.205–1.273) at 6 h after surgery, which can indicate that it can serve as an independent predictor of AKI (Fig. 3). The results of multivariate logistic regression analysis after the inclusion of clinical indicators were still statistically significant (Fig. 4). ROC analysis demonstrated that the AUC (0.807) reached its maximum at 6 h post-surgery, with high sensitivity (*p* < 0.001). A parallel ROC assessment of peri-operative transferrin change yielded a maximal AUC (0.763) at the identical 6-hour time point, underscoring its robust predictive value for AKI at this juncture.(Figures 2B and 6B).

### Hepcidin/uCr

Hepcidin/uCr levels showed an increased trend post-surgery in both non-AKI and AKI groups, which the non-AKI group increased more obviously. The differences between different time points and groups were statistically significant (Table 2; Fig. 1C). Univariate logistic regression analysis indicated that hepcidin/uCr had the maximum difference OR of 0.862 (0.851–0.876) at 0 h post-surgery, with statistical significance (Fig. 3). Multivariate logistic regression showed minimal differences after adjusting for confounders (Fig. 4). Hepcidin/uCr conforms as an independent risk factor for AKI post-CPB. Additionally, ROC analysis revealed that both the urinary hepcidin/uCr at 0 h post-surgery (AUC = 0.894) and the perioperative change from baseline (AUC = 0.799) achieved the highest area under the curve, with favourable sensitivity and specificity, indicating strong predictive performance for AKI at this time point (Figs. 2C and 6C).

### The combined predictive biomarkers for AKI and the severity of kidney injury

**Fig. 5:**
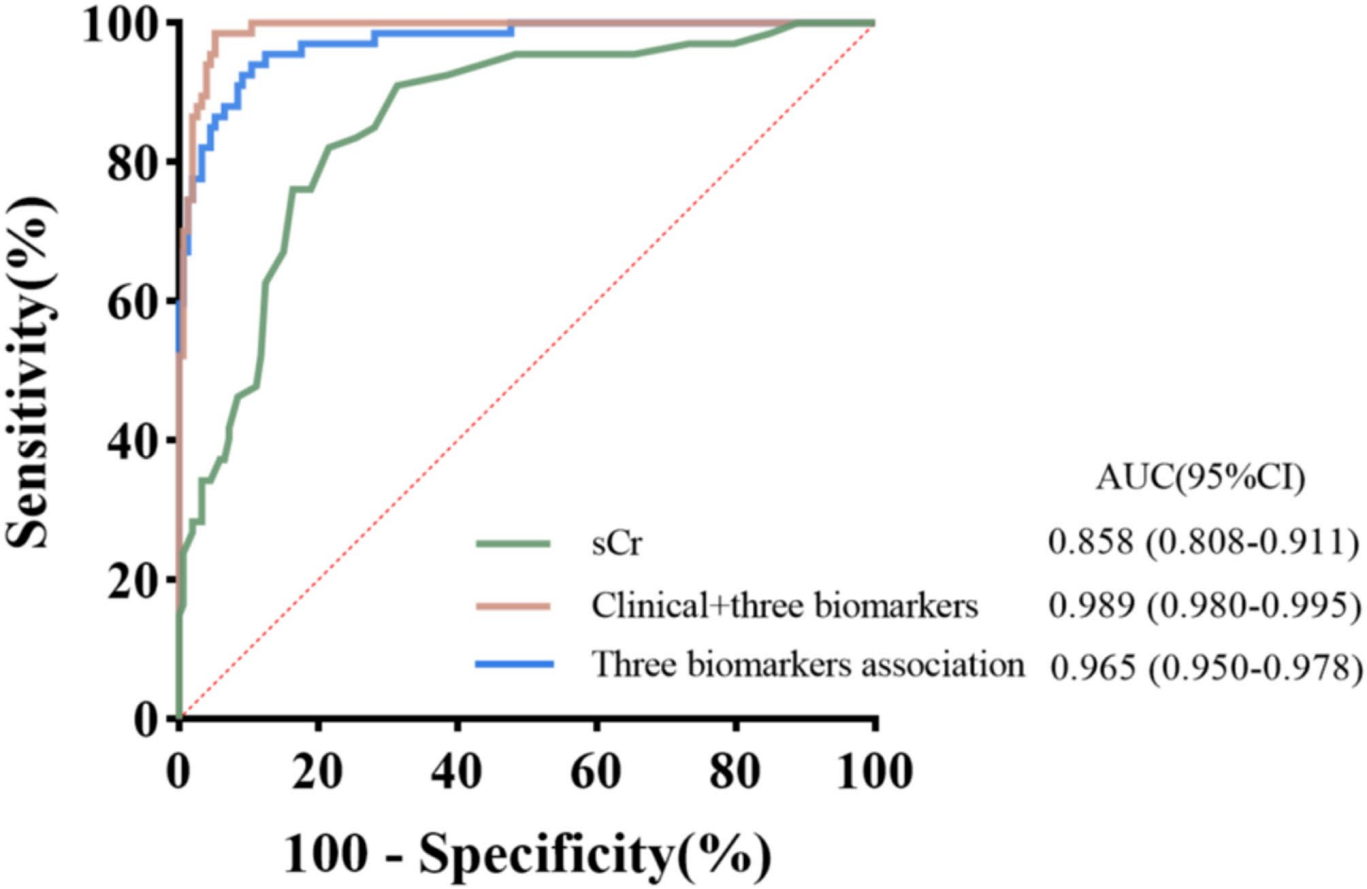
ROC analysis of combined biomarkers and sCr The ROC analysis was conducted to evaluate the predictive ability for AKI occurrence using sCr, a combination of three biomarkers, and a clinical combination of biomarkers, followed by a comparison of the results.sCr, serum creatinine; AUC, area under the curve; 95%CI, 95% Confidence interva

Combined the optimal time points of three iron-related factors to predict AKI—serum ferritin at 6 h post-surgery, transferrin/uCr at 6 h post-surgery, and hepcidin/uCr at 0 h post-surgery, we found that the ROC analysis revealed a significant increase in the AUC, sensitivity, and specificity. This greatly enhanced the predictive performance and improved the model’s effectiveness with the compared to sCr model. Furthermore, incorporating clinical indicators (postoperative creatinine, RACHS-1, aortic cross-clamp time, and postoperative eGFR) further boosted the predictive capability for AKI (Fig. 5). Multivariate logistic regression analysis confirmed that these three biomarkers are independent predictors of AKI (Fig. 4). When stratifying AKI patients according to the KDIGO criteria and testing the predictive models for each AKI stage, both models demonstrated strong predictive abilities. (Table 3).

**Fig. 6:**
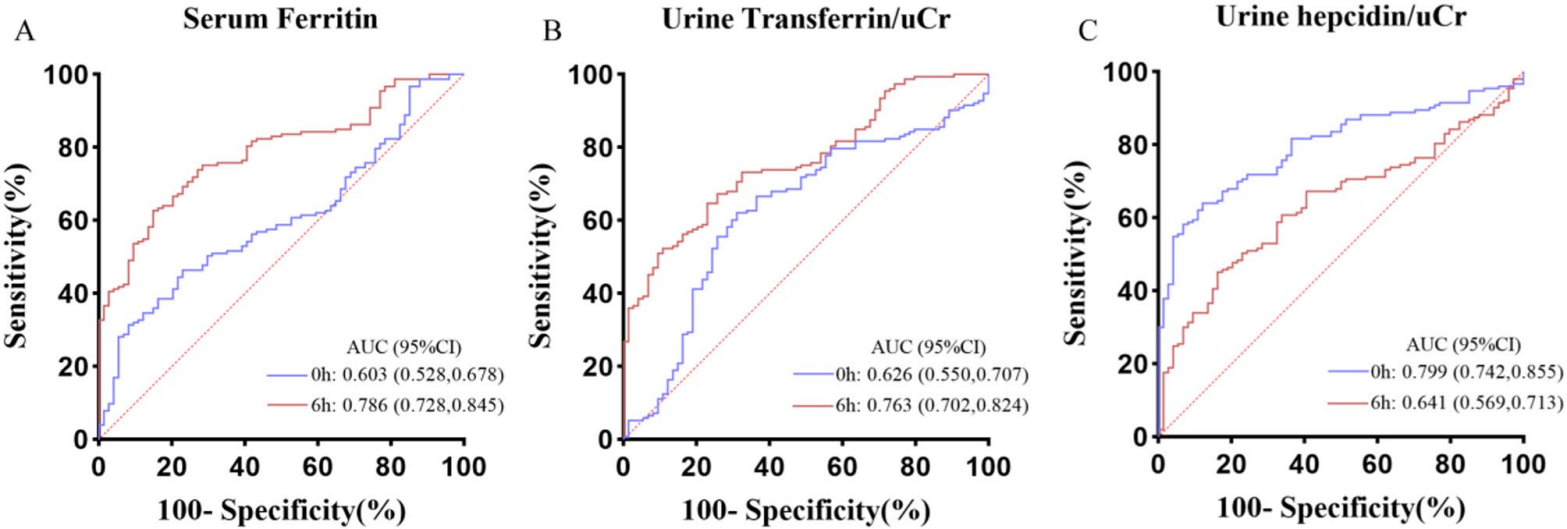
ROC analysis of biomarker change scores across serial peri-operative time points. To evaluate the discriminatory capacity of peri-operative changes in serum ferritin, urinary transferrin/uCr, and hepcidin/uCr for AKI, ROC analyses were performed across serial post-surgical time points. AUC, area under the curve; 95%CI, 95% Confidence intervalA.ROC analysis of postoperative 0h and 6h serum ferritin change, B.ROC analysis of postoperative 0h and 6h urinary transferrin/uCr change, C.ROC analysis of postoperative 0h and 6h hepcidin/uCr change

**Table 3:** Combined biomarkers ROC analysis for predicting AKI severity levels

	AKI Stage	AUC (95%CI)	*P*-value	sensitivity	specificity
Three biomarkers	AKI Stage 1	0.894 (0.851–0.936)	0.001	0.94	0.768
	AKI Stage ≥ 2	0.877 (0.842–0.921)	0.001	0.96	0.639
Clincal+ Three biomarkers	AKI Stage 1	0.902 (0.863–0.941)	0.001	0.98	0.819
	AKI Stage ≥ 2	0.904 (0.863–0.938)	0.001	0.97	0.956

clinical indicators: postoperative creatinine, RACHS-1, aortic cross-clamp time, and postoperative eGFR

## Discussion

Our study primarily focuses on the early prediction and evaluation of acute kidney injury in children following cardiac surgery. Through early detection of iron-related parameters, we have demonstrated that ferritin, transferrin, and hepcidin possess significant early predictive value for the occurrence of AKI in children. These biomarkers can serve as independent predictors of AKI. The combined predictive capability of these three biomarkers shows superior ability and higher efficacy compared to serum creatinine. Integrating these with clinical indicators is also an effective method to enhance predictive performance, allowing for a more comprehensive and accurate early identification of AKI. In terms of assessing the severity of kidney injury, the combined prediction using biomarkers offers greater advantages and is particularly significant for the early identification of higher-grade AKI.

The physiology of the child kidney exhibits significant age-dependent developmental characteristics. After birth, the kidney undergoes a continuous process of structural and functional maturation that spans the entire childhood period [18,19]. The glomerular filtration rate of term neonates approaches adult levels by 2–3 years after birth, while the maturation of renal tubular function continues into adolescence [20–22]. This developmental immaturity markedly reduces functional reserve and reparative capacity, rendering infants and young children particularly vulnerable to AKI [23]. Significant inter-month heterogeneity in renal development exists during early life; our data confirm an inverse association between chronological age and AKI incidence. Consequently, age was included as a critical confounder in all subsequent multivariable models.

Ferritin, the primary intracellular iron storage protein, plays a central role in maintaining iron homeostasis [24, 25]. Ischemia-reperfusion injury and systemic inflammatory responses during CPB can increase labile iron release, exerting direct cytotoxicity on renal tubular epithelial and endothelial cells. As an acute-phase reactant, serum ferritin rises markedly under stress and mitigates oxidative damage by sequestering free iron and limiting reactive oxygen species generation [24, 26, 27]. This mechanism aligns with the post-operative ferritin peak observed in our cohort. Davis et al. reported that a low preoperative serum ferritin concentration was associated with an increased risk of post-operative acute renal failure [13]. Our findings extend this observation by confirming that post-operative serum ferritin is an independent predictor of AKI after CPB, corroborating results from a prospective cohort of 301 adults [28]. The observed odds ratio for serum ferritin < 1 implies a protective effect, whereby elevated ferritin attenuates oxidative injury through iron chelation, consistent with Devarajan’s ischaemic AKI model in which dysregulated iron metabolism exacerbates apoptosis [29]. Recent meta-analyses also support ferritin as a potential therapeutic target for AKI after cardiac surgery [26, 30, 31]. Recent meta-analyses further support ferritin as a potential therapeutic target for post-cardiac-surgery AKI. The present study addresses this knowledge gap and further confirms the predictive value of ferritin for AKI after CPB.

Transferrin (78kD), the primary iron transport protein synthesized by the liver, mediates the circulatory transport of iron ions by binding Fe³⁺. Cellular iron uptake occurs through transferrin receptor (TFR1 and TFR2)-mediated endocytosis. Glomerular-filtered transferrin is predominantly reabsorbed by the proximal tubule [32, 33]. In a nephrotoxic animal model, Alfredo G et al. demonstrated that tubular injury impairs transferrin reabsorption, rendering urinary transferrin a marker of renal damage [34]. In the present cohort, urinary transferrin declined post-operatively in the non-AKI group, whereas a modest increase was observed in the AKI group. However, the elevation of urinary transferrin in AKI patients cannot be definitively attributed to a glomerular or tubular origin. In studies of tubular dysfunction excluding significant glomerular injury, increased urinary transferrin showed no significant correlation with Egfr [35]. It has been proposed that decreased transferrin reabsorption during iron overload may serve as a protective mechanism to attenuate tubular iron burden [11, 35]. Compensatory changes, including downregulation of TFR expression and upregulation of ferroportin (FPN) expression in proximal tubule cells, have also been observed in CKD animal models [11]. Nevertheless, the dynamic changes and precise mechanisms of transferrin in ischemia-reperfusion AKI require further investigation.

Hepcidin, a 25-peptide iron-regulatory hormone, negatively regulates cellular iron release by binding to and internalizing FPN. Under inflammatory conditions, elevated hepcidin levels suppress intestinal iron absorption and macrophage iron recycling [28, 34, 36]. In the present study, hepcidin levels were significantly higher in the non-AKI group than in the AKI group, and regression analysis confirmed it as a protective factor. Scindia et al. demonstrated in a murine ischaemia–reperfusion injury model that elevated serum hepcidin confers renoprotection, and exogenous hepcidin administration restores iron homeostasis and attenuates renal injury [19, 37, 38].These findings align with our results and underscore the diagnostic relevance of hepcidin in early AKI detection. To our knowledge, this is the first report to characterise the dynamic trajectories of serum hepcidin and their association with AKI following cardiac surgery in pediatric population. Although recent adult cohorts have observed similar trends, urinary hepcidin dynamics and the underlying regulatory mechanisms in AKI remain to be elucidated.

This study primarily examines the early postoperative predictive value of three combined factors for AKI, and postoperative differences constitute the central focus of our analysis. Nevertheless, we inevitably observed that pre-operative biomarker concentrations also differed between the AKI and non-AKI groups.We contend that these preoperative disparities reflect underlying iron-metabolic derangements that may serve as a critical mechanistic link among baseline disease severity, chronic cardiorenal injury, and subsequent postoperative AKI.The risk of postoperative AKI is therefore rooted, even before surgery, in the child’s underlying disease burden and pre-existing iron-metabolic imbalance.Pre-operative differences delineate a susceptible phenotype, whereas post-operative differences capture the magnitude of the injurious insult.To address this, we used perioperative changes in biomarkers to predict AKI, which yielded favorable results. This finding substantiates the robustness of our overall conclusions.

This study innovatively integrates three biomarkers from a comprehensive perspective of iron metabolism. Although serum ferritin has lower iron-carrying capacity than transferrin, it is indispensable for iron storage and buffering [20, 21]. Renal iron metabolism exhibits segmental heterogeneity, and iron overload can downregulate TFR1 expression in proximal tubules [22, 39]. Elevated circulating iron stimulates hepatic hepcidin synthesis, maintaining homeostasis through negative feedback regulation of FPN. Combined monitoring of the dynamic changes in these three markers can more sensitively reflect the degree of renal injury and iron homeostasis imbalance. The combined detection of biomarkers with clinical parameters is of greater diagnostic necessity.

Although derangements of iron metabolism have been linked to adult AKI [25], this is the first study to systematically evaluate serum ferritin, transferrin, and hepcidin for the prediction of AKI following cardiac surgery in pediatric population. We provide the initial demonstration, in a paediatric cohort, of both the independent and combined predictive performance of these three iron-related biomarkers, thereby addressing a critical gap in paediatric research and establishing a model tailored to this population. Furthermore, the postoperative dynamic trajectories of these biomarkers furnish clinical evidence supporting the mechanistic pathway linking cardiac surgery to iron-metabolic disruption and subsequent renal injury. Integration of biomarkers with established clinical variables overcomes the limitations of single-marker approaches and lays the groundwork for the future development of clinical decision-support tools. Iron-related proteins outperform conventional serum creatinine for early detection of AKI in pediatric population, permitting earlier recognition and providing a therapeutic window for timely intervention. Exogenous supplementation of hepcidin or ferritin may represent a novel prophylactic strategy and could emerge as a prospective therapeutic target for AKI in this age group.

However, this study also has certain limitations. The single-center design and limited sample size, especially the small number of stage 3 AKI cases, may constrain statistical power and generalizability of conclusions. Future multicenter large-sample validation is warranted. Participants were restricted to children aged 1–36 months; neonates and older children were under-represented. Future studies should encompass a broader age spectrum and establish age-stratified predictive thresholds. Although predictive associations were demonstrated, causal mechanisms linking the biomarkers to AKI require further elucidation through molecular and functional studies. Whether early prediction translates into improved outcomes when coupled with prophylactic interventions remains unknown and merits prospective interventional investigation. In addition, preoperative levels of the three biomarkers differed between groups. Although perioperative change scores were incorporated to refine prediction, residual confounding—particularly from unmeasured indices of baseline disease severity—cannot be fully excluded. Subsequent investigations should incorporate more granular perioperative and disease-specific covariates to minimise confounding.

## Conclusions

Ferritin, transferrin, and hepcidin exhibit significant early predictive value for ischemia-reperfusion-related AKI following cardiac surgery in children. and serve as independent predictors. Integrating these three biomarkers significantly enhances AKI predictive efficacy, outperforming the traditional serum creatinine indicator. Integrating biomarkers with established clinical parameters further enhances the early detection of postoperative AKI. This combined approach remains effective for stratifying AKI severity. These findings provide an iron-metabolism-centred theoretical framework for early AKI warning and for the development of targeted interventions in paediatric cardiac surgery.

## Data Availability

No datasets were generated or analysed during the current study.

## Abbreviations

AKI: Acute kidney injury
IR: Ischemia-reperfusion
CPB: Cardiopulmonary bypass
AUC: Area under the curve
ROC: Receiver operating characteristic
CHD: Congenital heart disease
ICU: Intensive care unit
ROS: Reactive oxygen species
OR: Odds ratios
CTA: Computed tomography angiography
BUN: Blood urea nitrogen
eGFR: estimated glomerular filtration rate
DHCA: Deep hypothermic circulatory arrest
RACHS-1: Risk Adjustment for Congenital Heart Surgery-1
sCr: Serum creatinine
KDIGO: Kidney Disease: Improving Global Outcomes
ELISA: Enzyme-linked immunosorbent assay
FPN: Ferroportin
TFR: Transferrin Receptor
